# (4′-Ethynyl-2,2′:6′,2′′-terpyridine)(2,2′:6′,2′′-terpyridine)­ruthenium(II) bis­(hexa­fluoridophosphate) acetonitrile disolvate

**DOI:** 10.1107/S1600536812051227

**Published:** 2013-01-09

**Authors:** Weizhong Chen, Francisca N. Rein, Brian L. Scott, Reginaldo C. Rocha

**Affiliations:** a Los Alamos National Laboratory, Los Alamos, NM 87545, USA

## Abstract

The title heteroleptic *bis­*-terpyridine complex, [Ru(C_15_H_11_N_3_)(C_17_H_11_N_3_)](PF_6_)_2_·2CH_3_CN, crystallized from an acetonitrile solution as a salt containing two hexa­fluoridophosphate counter-ions and two acetonitrile solvent mol­ecules. The Ru^II^ atom has a distorted octa­hedral geometry due to the restricted bite angle [157.7 (3)°] of the two *mer*-arranged *N*,*N*′,*N*′′-tridendate ligands, *viz.* 2,2′:6′,2′′-terpyridine (tpy) and 4′-ethynyl-2,2′:6′,2′′-terpyridine (tpy′), which are essentially perpendicular to each other, with a dihedral angle of 87.75 (12)° between their terpyridyl planes. The rod-like acetyl­ene group lies in the same plane as its adjacent terpyridyl moiety, with a maximum deviation of only 0.071 (11) Å from coplanarity with the pyridine rings. The mean Ru—N bond length involving the outer N atoms *trans* to each other is 2.069 (6) Å at tpy and 2.070 (6) Å at tpy′. The Ru—N bond length involving the central N atom is 1.964 (6) Å at tpy and 1.967 (6) Å at tpy′. Two of the three counter anions were refined as half-occupied.

## Related literature

For the crystal structure of a Ru^II^–terpyridine complex containing the {Ru(tpy–C C)} fragment, see: Ruben *et al.* (2008 ▶). For a comparative discussion, see the *Comment* section in the Supplementary materials. For bond lengths and angles in related tpy complexes, see: Lashgari *et al.* (1999 ▶); Scudder *et al.* (2005 ▶). For the preparation of the starting materials, see: Benniston *et al.* (2005 ▶); Grosshenny *et al.* (1997 ▶); Sullivan *et al.* (1980 ▶); Ziessel *et al.* (2004 ▶). For general properties of this complex and related systems, see: Grosshenny *et al.* (1996 ▶); Hammarström & Johansson (2010 ▶); Ruther *et al.* (2011 ▶); Ziessel *et al.* (2004 ▶).
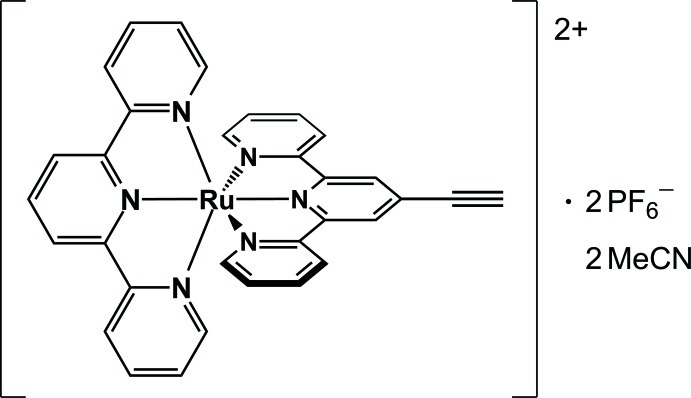


## Experimental

### 

#### Crystal data

[Ru(C_15_H_11_N_3_)(C_17_H_11_N_3_)](PF_6_)_2_·2C_2_H_3_N
*M* *_r_* = 963.67
Triclinic, 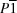

*a* = 8.704 (2) Å
*b* = 8.860 (2) Å
*c* = 27.277 (7) Å
α = 96.876 (4)°
β = 95.619 (3)°
γ = 93.023 (3)°
*V* = 2073.9 (10) Å^3^
*Z* = 2
Mo *K*α radiation
μ = 0.55 mm^−1^
*T* = 140 K
0.10 × 0.08 × 0.06 mm

#### Data collection

Bruker D8 with APEXII CCD diffractometer
Absorption correction: multi-scan (*SADABS*; Bruker, 2007 ▶) *T*_min_ = 0.947, *T*_max_ = 0.968
19870 measured reflections
7496 independent reflections
5137 reflections with *I* > 2σ(*I*)
*R*_int_ = 0.081

#### Refinement

*R*[*F* ^2^ > 2σ(*F* ^2^)] = 0.084
*wR*(*F* ^2^) = 0.228
*S* = 1.25
7496 reflections
596 parameters
H-atom parameters constrained
Δρ_max_ = 1.75 e Å^−3^
Δρ_min_ = −0.94 e Å^−3^

### 

Data collection: *APEX2* (Bruker, 2007 ▶); cell refinement: *SAINT-Plus* (Bruker, 2007 ▶); data reduction: *SAINT-Plus*; program(s) used to solve structure: *SHELXS97* (Sheldrick, 2008 ▶); program(s) used to refine structure: *SHELXL97* (Sheldrick, 2008 ▶); molecular graphics: *SHELXTL* (Sheldrick, 2008 ▶); software used to prepare material for publication: *publCIF* (Westrip, 2010 ▶).

## Supplementary Material

Crystal structure: contains datablock(s) I, global. DOI: 10.1107/S1600536812051227/sj5287sup1.cif

Structure factors: contains datablock(s) I. DOI: 10.1107/S1600536812051227/sj5287Isup2.hkl

Additional supplementary materials:  crystallographic information; 3D view; checkCIF report

## Figures and Tables

**Fig. 1.**
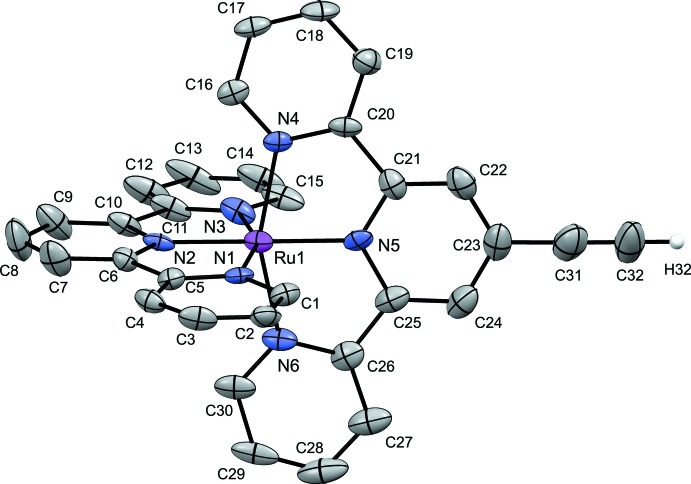
Figure 1. The single-crystal structure of the cation (I) in [RuII(tpy)(tpy')](PF6)2×2MeCN. Displacement ellipsoids are drawn at the 50% probability level. Except for H32, H atoms are omitted for clarity.

## supplementary crystallographic information

### Comment

The compound [Ru^II^(tpy)(tpy')](PF_6_)_2_×2MeCN crystallized in the triclinic space group (*P*1) from an acetonitrile solution. The crystal structure of its dication [Ru(tpy)(tpy')]^2+^ (I) is reported here for the first time, despite its well demonstrated relevance as a metallo-synthon unit into the construction of alkyne-bridged polyad arrays with optical/electronic applications (for example, see: Benniston *et al.*, 2005; Grosshenny *et al.*, 1996; Ziessel *et al.*, 2004) and, more recently, as interesting precursors to optically/electrochemically active interfacial assemblies *via* surface click chemistry at the alkynyl group (for example, see: Ruther *et al.*, 2011).

The only other crystallographically characterized compound featuring the {Ru(tpy–C≡C)} fragment is the homoleptic complex [Ru^II^(tpy")_2_](PF_6_)_2_ (II; tpy" = *S*-(4-[2,2':6',2"]terpyridin-4'-ylethynyl-phenyl ester), which was applied in studies of molecular electronics involving charge transport through single molecules (II) in break-junction configurations (Ruben *et al.*, 2008). In this case, the compound also crystallized in the triclinic space group (*P*1). In II, the two elongated ligands pointed along the long axis of the complex, with only slight distortion across the metal center (N–Ru–N angle: 178.9 (4)°). The mean Ru—N bond distances (1.966 (8) Å for the central nitrogen and 2.066 (10) Å for the outer nitrogen atoms *trans* to each other) as well as the tpy" bite angles (158.3 (4)°) are very similar to those observed for I.

These distances and angles are also in very good agreement with typical values reported for [Ru(tpy)_2_]^2+^ (*e.g.*, Lashgari *et al.*, 1999; Scudder *et al.*, 2005). The bite angle of terpyridines is well known to be far from the ideal 180° due to the unfavorable *N,N,N* geometric configuration of the *mer*-terdentate ligand (Hammarström & Johansson, 2010). In I, the two terpyridyl ligands are approximately planar, with only a slight bending towards the outer ring atoms (maximum deviation from planarity: 0.093 (10) Å for atom C13 at tpy and 0.110 (8) Å for atom C30 at tpy'). The acetylenic group (–C31≡C32–H32) lies along the main axis passing through the metal center as well as in the same plane as its adjacent terpyridyl moiety, with a maximum deviation of only 0.071 (11) Å (C32) from coplanarity. The length of the triple bond between C31 and C32 is 1.175 (13) Å.

### Experimental

The compound [Ru(tpy)(tpy')](PF_6_)_2_ was prepared from the precursor [Ru(tpy)(tpy'-TMS)](PF_6_)_2_ (tpy'-TMS = 4'-trimethylsilylethynyl-(2,2':6',2"-terpy)) as described in the literature (Benniston *et al.*, 2005). Also synthesized according to reported procedures were the starting materials Ru(tpy)Cl_3_ (Sullivan *et al.*, 1980) and tpy'-TMS (Grosshenny *et al.*, 1997). The identity of the cation [Ru(tpy)(tpy')]^2+^ (I) in solution was also confirmed by electrochemical and spectroscopic methods. Single crystals suitable for X-ray analysis were grown by slow diffusion of Et_2_O into MeCN solutions of [Ru(tpy)(tpy')](PF_6_)_2_ in a long thin tube.

### Refinement

The structure was solved by using direct methods and difference Fourier techniques. All hydrogen atom positions were idealized, and rode on the atom they were attached to. The final refinement included anisotropic temperature factors on all non-hydrogen atoms.

Two of the hexafluorophosphate anions had very large temperature factors when compared to the third. Other characterization by nuclear magnetic resonance, electronic absorption spectroscopy, and electrochemical techniques clearly support the oxidation state +2 for the Ru center. As a result, the two hexafluorophosphate anions were refined at one-half occupancy.

### Crystal data

**Table:**

[Ru(C_15_H_11_N_3_)(C_17_H_11_N_3_)](PF_6_)_2_·2C_2_H_3_N	*Z* = 2
*M**_r_* = 963.67	*F*(000) = 964
Triclinic, *P*1	*D*_x_ = 1.543 Mg m^−^^3^
Hall symbol: -P 1	Mo *K*α radiation, λ = 0.71073 Å
*a* = 8.704 (2) Å	Cell parameters from 1822 reflections
*b* = 8.860 (2) Å	θ = 4.7–41.0°
*c* = 27.277 (7) Å	µ = 0.55 mm^−^^1^
α = 96.876 (4)°	*T* = 140 K
β = 95.619 (3)°	Block, orange
γ = 93.023 (3)°	0.10 × 0.08 × 0.06 mm
*V* = 2073.9 (10) Å^3^	

### Data collection

**Table:**

Bruker D8 with APEXII CCD diffractometer	7496 independent reflections
Radiation source: fine-focus sealed tube	5137 reflections with *I* > 2σ(*I*)
Graphite monochromator	*R*_int_ = 0.081
ω scans	θ_max_ = 25.3°, θ_min_ = 2.3°
Absorption correction: multi-scan (*SADABS*; Bruker, 2007)	*h* = −10→10
*T*_min_ = 0.947, *T*_max_ = 0.968	*k* = −10→10
19870 measured reflections	*l* = −32→32

### Refinement

**Table:**

Refinement on *F*^2^	Primary atom site location: structure-invariant direct methods
Least-squares matrix: full	Secondary atom site location: difference Fourier map
*R*[*F*^2^ > 2σ(*F*^2^)] = 0.084	Hydrogen site location: inferred from neighbouring sites
*wR*(*F*^2^) = 0.228	H-atom parameters constrained
*S* = 1.25	*w* = 1/[σ^2^(*F*_o_^2^) + (0.1*P*)^2^] where *P* = (*F*_o_^2^ + 2*F*_c_^2^)/3
7496 reflections	(Δ/σ)_max_ < 0.001
596 parameters	Δρ_max_ = 1.75 e Å^−^^3^
0 restraints	Δρ_min_ = −0.94 e Å^−^^3^

### Special details

**Table:**

Geometry. All e.s.d.'s (except the e.s.d. in the dihedral angle between two l.s. planes) are estimated using the full covariance matrix. The cell e.s.d.'s are taken into account individually in the estimation of e.s.d.'s in distances, angles and torsion angles; correlations between e.s.d.'s in cell parameters are only used when they are defined by crystal symmetry. An approximate (isotropic) treatment of cell e.s.d.'s is used for estimating e.s.d.'s involving l.s. planes.
Refinement. Refinement of *F*^2^ against ALL reflections. The weighted *R*-factor *wR* and goodness of fit *S* are based on *F*^2^, conventional *R*-factors *R* are based on *F*, with *F* set to zero for negative *F*^2^. The threshold expression of *F*^2^ > σ(*F*^2^) is used only for calculating *R*-factors(gt) *etc*. and is not relevant to the choice of reflections for refinement. *R*-factors based on *F*^2^ are statistically about twice as large as those based on *F*, and *R*- factors based on ALL data will be even larger.Note: Two of the hexafluorophosphate anions had very large temperature factors when compared to the third. Other characterization by nuclear magnetic resonance, electronic absorption spectroscopy, and electrochemical techniques clearly support the oxidation state +2 for the Ru center. As a result, the two hexafluorophosphate anions were refined at one-half occupancy.

### Fractional atomic coordinates and isotropic or equivalent isotropic displacement parameters (Å^2^)

**Table:**

	*x*	*y*	*z*	*U*_iso_*/*U*_eq_	Occ. (<1)
Ru1	0.60446 (7)	0.66280 (7)	0.71976 (2)	0.0229 (2)	
P1	0.5105 (3)	0.2494 (3)	0.86893 (9)	0.0406 (6)	
P2	0.0665 (4)	0.3760 (5)	0.58567 (15)	0.0223 (9)	0.50
P3	0.3376 (7)	0.9331 (5)	0.52521 (18)	0.0460 (13)	0.50
F1	0.4111 (7)	0.3779 (7)	0.8487 (2)	0.0662 (17)	
F2	0.3720 (7)	0.1889 (8)	0.8960 (2)	0.083 (2)	
F3	0.6136 (8)	0.1235 (7)	0.8895 (3)	0.087 (2)	
F4	0.6488 (6)	0.3082 (6)	0.8399 (2)	0.0627 (16)	
F5	0.5731 (8)	0.3658 (7)	0.9169 (2)	0.080 (2)	
F6	0.4524 (7)	0.1353 (6)	0.8207 (2)	0.0699 (18)	
F7	0.2344 (13)	0.4242 (15)	0.5732 (4)	0.071 (4)	0.50
F8	0.0213 (12)	0.3542 (11)	0.5289 (3)	0.048 (3)	0.50
F9	−0.0984 (14)	0.3277 (16)	0.5993 (4)	0.074 (4)	0.50
F10	0.1149 (13)	0.3953 (11)	0.6387 (3)	0.048 (3)	0.50
F11	0.0255 (16)	0.5450 (15)	0.5929 (5)	0.086 (4)	0.50
F12	0.1067 (14)	0.2015 (14)	0.5798 (5)	0.078 (4)	0.50
F13	0.2819 (11)	0.7699 (11)	0.5355 (4)	0.052 (3)	0.50
F14	0.4594 (13)	0.9411 (12)	0.5740 (4)	0.055 (3)	0.50
F15	0.2102 (13)	0.9903 (13)	0.5575 (4)	0.061 (3)	0.50
F16	0.3906 (14)	1.1053 (11)	0.5201 (4)	0.051 (3)	0.50
F17	0.2281 (13)	0.9269 (11)	0.4820 (5)	0.066 (4)	0.50
F18	0.4732 (18)	0.8751 (13)	0.4927 (5)	0.080 (4)	0.50
N1	0.6951 (6)	0.8758 (7)	0.7100 (2)	0.0218 (14)	
N2	0.6314 (7)	0.6327 (6)	0.6487 (2)	0.0223 (14)	
N3	0.5163 (7)	0.4394 (7)	0.7002 (3)	0.0306 (16)	
N4	0.8136 (7)	0.5925 (6)	0.7502 (2)	0.0212 (14)	
N5	0.5824 (7)	0.6975 (6)	0.7912 (2)	0.0241 (14)	
N6	0.3870 (7)	0.7435 (7)	0.7184 (3)	0.0273 (15)	
N7	0.9608 (12)	−0.0287 (12)	0.8456 (4)	0.082 (3)	
N8	0.0331 (14)	0.6614 (13)	0.9563 (4)	0.088 (3)	
C1	0.7199 (8)	1.0006 (9)	0.7444 (3)	0.0273 (18)	
H1	0.6942	0.9936	0.7764	0.033*	
C2	0.7826 (9)	1.1392 (9)	0.7334 (3)	0.032 (2)	
H2	0.7969	1.2236	0.7575	0.039*	
C3	0.8230 (9)	1.1495 (8)	0.6865 (3)	0.032 (2)	
H3	0.8692	1.2396	0.6788	0.039*	
C4	0.7941 (9)	1.0242 (9)	0.6506 (3)	0.0291 (19)	
H4	0.8165	1.0314	0.6183	0.035*	
C5	0.7316 (8)	0.8874 (8)	0.6628 (3)	0.0234 (17)	
C6	0.6973 (9)	0.7496 (9)	0.6277 (3)	0.0251 (17)	
C7	0.7184 (11)	0.7255 (10)	0.5781 (3)	0.042 (2)	
H7	0.7616	0.8050	0.5635	0.050*	
C8	0.6785 (13)	0.5893 (11)	0.5496 (3)	0.056 (3)	
H8	0.6959	0.5754	0.5163	0.067*	
C9	0.6102 (12)	0.4699 (10)	0.5717 (3)	0.048 (3)	
H9	0.5808	0.3758	0.5533	0.058*	
C10	0.5881 (9)	0.4966 (8)	0.6215 (3)	0.0294 (19)	
C11	0.5205 (9)	0.3899 (9)	0.6516 (3)	0.034 (2)	
C12	0.4598 (11)	0.2427 (9)	0.6311 (4)	0.051 (3)	
H12	0.4632	0.2099	0.5976	0.061*	
C13	0.3950 (11)	0.1473 (10)	0.6612 (4)	0.061 (3)	
H13	0.3528	0.0503	0.6484	0.073*	
C14	0.3951 (10)	0.2002 (10)	0.7107 (4)	0.050 (3)	
H14	0.3551	0.1369	0.7319	0.059*	
C15	0.4541 (9)	0.3471 (9)	0.7297 (4)	0.038 (2)	
H15	0.4505	0.3815	0.7631	0.046*	
C16	0.9277 (8)	0.5350 (8)	0.7264 (3)	0.0251 (17)	
H16	0.9201	0.5297	0.6920	0.030*	
C17	1.0570 (9)	0.4828 (9)	0.7508 (3)	0.0312 (19)	
H17	1.1334	0.4413	0.7328	0.037*	
C18	1.0729 (9)	0.4920 (9)	0.8014 (3)	0.036 (2)	
H18	1.1600	0.4586	0.8184	0.043*	
C19	0.9558 (9)	0.5523 (9)	0.8265 (3)	0.033 (2)	
H19	0.9635	0.5599	0.8610	0.040*	
C20	0.8264 (8)	0.6019 (8)	0.8006 (3)	0.0252 (18)	
C21	0.6948 (10)	0.6671 (9)	0.8251 (3)	0.0304 (19)	
C22	0.6838 (11)	0.6954 (9)	0.8747 (3)	0.038 (2)	
H22	0.7631	0.6723	0.8975	0.045*	
C23	0.5526 (12)	0.7590 (9)	0.8908 (3)	0.041 (2)	
C24	0.4340 (11)	0.7933 (9)	0.8559 (3)	0.041 (2)	
H24	0.3463	0.8378	0.8662	0.049*	
C25	0.4506 (9)	0.7593 (9)	0.8058 (3)	0.033 (2)	
C26	0.3421 (9)	0.7893 (9)	0.7645 (3)	0.032 (2)	
C27	0.2062 (9)	0.8608 (9)	0.7703 (4)	0.042 (2)	
H27	0.1808	0.8962	0.8018	0.051*	
C28	0.1083 (10)	0.8787 (10)	0.7282 (4)	0.053 (3)	
H28	0.0161	0.9257	0.7312	0.063*	
C29	0.1501 (9)	0.8255 (9)	0.6820 (4)	0.041 (2)	
H29	0.0854	0.8357	0.6536	0.049*	
C30	0.2887 (9)	0.7569 (9)	0.6782 (4)	0.035 (2)	
H30	0.3144	0.7191	0.6470	0.042*	
C31	0.5415 (13)	0.7943 (11)	0.9431 (4)	0.058 (3)	
C32	0.5330 (16)	0.8213 (12)	0.9860 (4)	0.072 (4)	
H32	0.5263	0.8427	1.0199	0.087*	
C33	0.9607 (15)	0.0376 (19)	0.8804 (5)	0.104 (6)	
C34	0.958 (2)	0.113 (4)	0.9314 (8)	0.36 (3)	
H34A	0.8897	0.0544	0.9486	0.541*	
H34B	0.9217	0.2132	0.9303	0.541*	
H34C	1.0603	0.1207	0.9485	0.541*	
C35	0.1365 (14)	0.5885 (14)	0.9648 (4)	0.063 (3)	
C36	0.2593 (15)	0.4921 (15)	0.9767 (5)	0.084 (4)	
H36A	0.3438	0.5523	0.9962	0.126*	
H36B	0.2941	0.4450	0.9465	0.126*	
H36C	0.2220	0.4148	0.9952	0.126*	

### Atomic displacement parameters (Å^2^)

**Table:**

	*U*^11^	*U*^22^	*U*^33^	*U*^12^	*U*^13^	*U*^23^
Ru1	0.0183 (3)	0.0179 (3)	0.0337 (4)	−0.0009 (2)	0.0022 (3)	0.0091 (3)
P1	0.0455 (14)	0.0296 (13)	0.0456 (15)	0.0006 (11)	−0.0040 (11)	0.0080 (11)
P2	0.0167 (19)	0.025 (2)	0.027 (2)	0.0045 (16)	0.0036 (16)	0.0105 (17)
P3	0.072 (4)	0.029 (3)	0.035 (3)	−0.003 (2)	0.010 (3)	−0.005 (2)
F1	0.055 (4)	0.064 (4)	0.086 (4)	0.028 (3)	0.006 (3)	0.025 (3)
F2	0.067 (4)	0.104 (6)	0.080 (5)	−0.027 (4)	0.019 (3)	0.029 (4)
F3	0.072 (5)	0.057 (4)	0.134 (6)	0.011 (3)	−0.018 (4)	0.044 (4)
F4	0.054 (4)	0.055 (4)	0.083 (4)	−0.006 (3)	0.013 (3)	0.021 (3)
F5	0.111 (6)	0.068 (4)	0.052 (4)	−0.030 (4)	−0.008 (4)	0.002 (3)
F6	0.092 (5)	0.045 (4)	0.065 (4)	−0.021 (3)	0.003 (3)	−0.007 (3)
F7	0.055 (8)	0.095 (10)	0.067 (8)	−0.012 (7)	−0.004 (6)	0.038 (7)
F8	0.076 (8)	0.055 (7)	0.009 (5)	−0.040 (6)	0.004 (4)	0.008 (4)
F9	0.062 (8)	0.101 (10)	0.077 (9)	0.020 (7)	0.026 (7)	0.056 (8)
F10	0.083 (8)	0.033 (6)	0.036 (6)	0.015 (5)	0.047 (5)	0.001 (4)
F11	0.085 (10)	0.062 (9)	0.099 (10)	0.021 (7)	−0.043 (8)	0.001 (7)
F12	0.067 (8)	0.060 (8)	0.098 (10)	0.021 (7)	−0.024 (7)	−0.008 (7)
F13	0.035 (6)	0.041 (6)	0.072 (8)	−0.027 (5)	0.016 (5)	−0.023 (5)
F14	0.073 (8)	0.049 (7)	0.047 (7)	0.030 (6)	0.006 (6)	0.006 (5)
F15	0.049 (7)	0.059 (8)	0.073 (8)	0.004 (6)	−0.006 (6)	0.013 (6)
F16	0.080 (9)	0.035 (6)	0.035 (6)	−0.021 (6)	−0.003 (6)	0.002 (5)
F17	0.060 (7)	0.020 (5)	0.109 (10)	−0.015 (5)	−0.032 (7)	0.019 (6)
F18	0.113 (12)	0.050 (8)	0.070 (9)	−0.029 (8)	0.033 (8)	−0.021 (6)
N1	0.014 (3)	0.018 (3)	0.034 (4)	0.006 (2)	−0.001 (3)	0.003 (3)
N2	0.017 (3)	0.013 (3)	0.036 (4)	−0.001 (2)	−0.008 (3)	0.008 (3)
N3	0.023 (4)	0.025 (4)	0.043 (4)	−0.009 (3)	−0.007 (3)	0.015 (3)
N4	0.019 (3)	0.011 (3)	0.032 (4)	−0.001 (2)	−0.002 (3)	0.001 (3)
N5	0.026 (3)	0.011 (3)	0.037 (4)	0.004 (3)	0.009 (3)	0.007 (3)
N6	0.015 (3)	0.020 (3)	0.048 (4)	−0.004 (3)	−0.001 (3)	0.012 (3)
N7	0.078 (7)	0.084 (8)	0.079 (8)	−0.020 (6)	0.031 (6)	−0.024 (6)
N8	0.096 (9)	0.098 (9)	0.065 (7)	0.019 (7)	−0.022 (6)	0.009 (6)
C1	0.022 (4)	0.022 (4)	0.036 (5)	0.003 (3)	0.002 (3)	0.000 (4)
C2	0.035 (5)	0.024 (5)	0.037 (5)	0.013 (4)	−0.002 (4)	0.001 (4)
C3	0.029 (4)	0.010 (4)	0.055 (6)	−0.004 (3)	−0.007 (4)	0.007 (4)
C4	0.025 (4)	0.028 (5)	0.036 (5)	−0.001 (3)	0.000 (4)	0.016 (4)
C5	0.020 (4)	0.021 (4)	0.030 (4)	0.003 (3)	0.004 (3)	0.004 (3)
C6	0.026 (4)	0.023 (4)	0.026 (4)	0.002 (3)	0.000 (3)	0.005 (3)
C7	0.063 (6)	0.024 (5)	0.039 (5)	−0.005 (4)	0.004 (5)	0.016 (4)
C8	0.100 (9)	0.043 (6)	0.022 (5)	0.004 (6)	−0.002 (5)	0.000 (4)
C9	0.080 (7)	0.021 (5)	0.037 (6)	−0.003 (5)	−0.023 (5)	0.003 (4)
C10	0.035 (5)	0.014 (4)	0.037 (5)	−0.003 (3)	−0.013 (4)	0.006 (3)
C11	0.030 (5)	0.014 (4)	0.057 (6)	−0.012 (3)	−0.015 (4)	0.018 (4)
C12	0.059 (6)	0.018 (5)	0.070 (7)	−0.007 (4)	−0.031 (5)	0.012 (4)
C13	0.056 (6)	0.024 (5)	0.096 (9)	−0.025 (5)	−0.045 (6)	0.035 (5)
C14	0.041 (6)	0.027 (5)	0.079 (8)	−0.015 (4)	−0.021 (5)	0.031 (5)
C15	0.017 (4)	0.033 (5)	0.066 (6)	−0.011 (4)	−0.010 (4)	0.027 (4)
C16	0.023 (4)	0.011 (4)	0.041 (5)	−0.005 (3)	0.006 (4)	0.003 (3)
C17	0.017 (4)	0.020 (4)	0.056 (6)	0.002 (3)	0.004 (4)	0.004 (4)
C18	0.018 (4)	0.029 (5)	0.059 (6)	0.005 (3)	−0.007 (4)	0.003 (4)
C19	0.037 (5)	0.028 (5)	0.033 (5)	0.005 (4)	−0.003 (4)	0.000 (4)
C20	0.021 (4)	0.013 (4)	0.043 (5)	0.000 (3)	−0.001 (4)	0.009 (3)
C21	0.038 (5)	0.024 (4)	0.028 (5)	−0.006 (4)	0.000 (4)	0.006 (3)
C22	0.052 (6)	0.027 (5)	0.035 (5)	−0.002 (4)	0.002 (4)	0.011 (4)
C23	0.071 (7)	0.020 (4)	0.036 (5)	0.000 (4)	0.019 (5)	0.006 (4)
C24	0.049 (6)	0.027 (5)	0.053 (6)	0.004 (4)	0.028 (5)	0.012 (4)
C25	0.028 (4)	0.021 (4)	0.053 (6)	0.001 (3)	0.015 (4)	0.010 (4)
C26	0.030 (4)	0.020 (4)	0.049 (5)	−0.004 (3)	0.015 (4)	0.013 (4)
C27	0.026 (5)	0.024 (5)	0.081 (7)	0.000 (4)	0.015 (5)	0.015 (5)
C28	0.024 (5)	0.030 (5)	0.111 (9)	0.003 (4)	0.017 (6)	0.025 (6)
C29	0.019 (4)	0.021 (5)	0.085 (7)	−0.008 (3)	−0.008 (5)	0.033 (5)
C30	0.026 (4)	0.015 (4)	0.066 (6)	−0.003 (3)	0.000 (4)	0.017 (4)
C31	0.083 (8)	0.039 (6)	0.058 (7)	0.019 (6)	0.028 (6)	0.011 (5)
C32	0.134 (12)	0.046 (7)	0.047 (7)	0.035 (7)	0.036 (7)	0.013 (5)
C33	0.055 (8)	0.147 (15)	0.089 (11)	0.011 (9)	−0.007 (7)	−0.058 (10)
C34	0.15 (2)	0.60 (6)	0.23 (3)	0.16 (3)	−0.099 (19)	−0.33 (3)
C35	0.072 (8)	0.077 (8)	0.036 (6)	0.026 (7)	−0.009 (5)	−0.003 (5)
C36	0.082 (10)	0.085 (10)	0.085 (9)	0.004 (8)	0.004 (7)	0.013 (7)

### Geometric parameters (Å, º)

**Table:**

Ru1—N2	1.964 (6)	C8—C9	1.411 (13)
Ru1—N5	1.967 (6)	C9—C10	1.385 (12)
Ru1—N6	2.057 (6)	C10—C11	1.461 (11)
Ru1—N1	2.064 (6)	C11—C12	1.410 (11)
Ru1—N3	2.073 (6)	C12—C13	1.384 (13)
Ru1—N4	2.083 (6)	C13—C14	1.374 (14)
P1—F2	1.577 (6)	C14—C15	1.394 (12)
P1—F6	1.583 (6)	C16—C17	1.381 (10)
P1—F1	1.585 (6)	C17—C18	1.364 (11)
P1—F3	1.588 (6)	C18—C19	1.378 (11)
P1—F5	1.596 (6)	C19—C20	1.389 (11)
P1—F4	1.602 (6)	C20—C21	1.489 (11)
P2—F10	1.453 (11)	C21—C22	1.360 (11)
P2—F8	1.547 (9)	C22—C23	1.386 (12)
P2—F11	1.551 (13)	C23—C24	1.404 (13)
P2—F9	1.568 (12)	C23—C31	1.438 (13)
P2—F7	1.581 (12)	C24—C25	1.388 (11)
P2—F12	1.597 (12)	C25—C26	1.457 (12)
P3—F17	1.435 (12)	C26—C27	1.385 (11)
P3—F15	1.551 (13)	C27—C28	1.390 (13)
P3—F13	1.567 (11)	C28—C29	1.381 (13)
P3—F16	1.597 (11)	C29—C30	1.387 (11)
P3—F14	1.610 (12)	C31—C32	1.175 (13)
P3—F18	1.613 (15)	C33—C34	1.47 (2)
F16—F18^i^	1.278 (16)	C35—C36	1.439 (16)
F18—F16^i^	1.278 (16)	C1—H1	0.93
N1—C1	1.355 (9)	C2—H2	0.93
N1—C5	1.370 (9)	C3—H3	0.93
N2—C10	1.353 (9)	C4—H4	0.93
N2—C6	1.368 (9)	C7—H7	0.93
N3—C15	1.344 (10)	C8—H8	0.93
N3—C11	1.350 (10)	C9—H9	0.93
N4—C16	1.330 (9)	C12—H12	0.93
N4—C20	1.361 (9)	C13—H13	0.93
N5—C21	1.338 (10)	C14—H14	0.93
N5—C25	1.370 (10)	C15—H15	0.93
N6—C30	1.344 (10)	C16—H16	0.93
N6—C26	1.373 (10)	C17—H17	0.93
N7—C33	1.054 (13)	C18—H18	0.93
N8—C35	1.157 (14)	C19—H19	0.93
C1—C2	1.395 (11)	C22—H22	0.93
C2—C3	1.371 (11)	C24—H24	0.93
C3—C4	1.383 (11)	C27—H27	0.93
C4—C5	1.392 (10)	C28—H28	0.93
C5—C6	1.456 (10)	C29—H29	0.93
C6—C7	1.376 (11)	C30—H30	0.93
C7—C8	1.363 (12)	C32—H32	0.93
			
N2—Ru1—N5	178.3 (3)	N2—C6—C7	118.3 (7)
N2—Ru1—N6	101.3 (3)	N2—C6—C5	112.5 (6)
N5—Ru1—N6	79.4 (3)	C7—C6—C5	129.1 (7)
N2—Ru1—N1	79.0 (2)	C8—C7—C6	122.4 (8)
N5—Ru1—N1	99.5 (2)	C7—C8—C9	118.6 (9)
N6—Ru1—N1	89.9 (2)	C10—C9—C8	118.2 (8)
N2—Ru1—N3	78.8 (3)	N2—C10—C9	121.4 (7)
N5—Ru1—N3	102.6 (3)	N2—C10—C11	111.3 (7)
N6—Ru1—N3	92.4 (2)	C9—C10—C11	127.3 (7)
N1—Ru1—N3	157.7 (3)	N3—C11—C12	121.0 (8)
N2—Ru1—N4	100.9 (2)	N3—C11—C10	117.0 (7)
N5—Ru1—N4	78.3 (2)	C12—C11—C10	122.1 (9)
N6—Ru1—N4	157.7 (3)	C13—C12—C11	119.6 (10)
N1—Ru1—N4	94.5 (2)	C14—C13—C12	118.0 (9)
N3—Ru1—N4	91.7 (2)	C13—C14—C15	121.1 (9)
F2—P1—F6	90.3 (4)	N3—C15—C14	120.6 (9)
F2—P1—F1	91.5 (4)	N4—C16—C17	122.4 (8)
F6—P1—F1	90.6 (4)	C18—C17—C16	120.1 (8)
F2—P1—F3	89.5 (4)	C17—C18—C19	118.0 (8)
F6—P1—F3	90.3 (4)	C18—C19—C20	120.4 (8)
F1—P1—F3	178.7 (4)	N4—C20—C19	120.6 (7)
F2—P1—F5	91.2 (4)	N4—C20—C21	115.9 (6)
F6—P1—F5	178.4 (4)	C19—C20—C21	123.5 (7)
F1—P1—F5	89.4 (4)	N5—C21—C22	122.1 (8)
F3—P1—F5	89.7 (4)	N5—C21—C20	110.8 (7)
F2—P1—F4	178.3 (4)	C22—C21—C20	127.0 (8)
F6—P1—F4	88.0 (3)	C21—C22—C23	119.0 (8)
F1—P1—F4	88.2 (3)	C22—C23—C24	119.8 (8)
F3—P1—F4	90.8 (4)	C22—C23—C31	119.7 (9)
F5—P1—F4	90.4 (4)	C24—C23—C31	120.5 (9)
F10—P2—F8	177.8 (6)	C25—C24—C23	118.6 (8)
F10—P2—F11	86.5 (6)	N5—C25—C24	120.0 (8)
F8—P2—F11	94.6 (7)	N5—C25—C26	113.5 (7)
F10—P2—F9	86.5 (6)	C24—C25—C26	126.4 (8)
F8—P2—F9	95.4 (6)	N6—C26—C27	121.9 (8)
F11—P2—F9	89.2 (8)	N6—C26—C25	114.4 (7)
F10—P2—F7	92.1 (6)	C27—C26—C25	123.7 (8)
F8—P2—F7	85.9 (6)	C26—C27—C28	118.8 (9)
F11—P2—F7	91.1 (8)	C29—C28—C27	119.0 (8)
F9—P2—F7	178.6 (6)	C28—C29—C30	119.9 (9)
F10—P2—F12	92.2 (6)	N6—C30—C29	121.8 (9)
F8—P2—F12	86.8 (6)	C32—C31—C23	179.1 (11)
F11—P2—F12	178.3 (8)	N7—C33—C34	173 (2)
F9—P2—F12	89.6 (7)	N8—C35—C36	176.8 (15)
F7—P2—F12	90.1 (7)	N1—C1—H1	119
F17—P3—F15	88.9 (8)	C2—C1—H1	119
F17—P3—F13	92.5 (6)	C1—C2—H2	121
F15—P3—F13	85.6 (6)	C3—C2—H2	120
F17—P3—F16	90.7 (6)	C2—C3—H3	121
F15—P3—F16	89.8 (7)	C4—C3—H3	120
F13—P3—F16	174.3 (6)	C3—C4—H4	120
F17—P3—F14	179.5 (8)	C5—C4—H4	120
F15—P3—F14	90.6 (6)	C6—C7—H7	119
F13—P3—F14	87.7 (6)	C8—C7—H7	119
F16—P3—F14	89.1 (6)	C7—C8—H8	121
F17—P3—F18	92.4 (8)	C9—C8—H8	121
F15—P3—F18	178.6 (8)	C8—C9—H9	121
F13—P3—F18	94.8 (6)	C10—C9—H9	121
F16—P3—F18	89.8 (6)	C11—C12—H12	120
F14—P3—F18	88.1 (7)	C13—C12—H12	120
F18^i^—F16—P3	115.0 (10)	C12—C13—H13	121
F16^i^—F18—P3	149.8 (10)	C14—C13—H13	121
C1—N1—C5	118.6 (6)	C13—C14—H14	120
C1—N1—Ru1	127.3 (5)	C15—C14—H14	119
C5—N1—Ru1	114.1 (5)	N3—C15—H15	120
C10—N2—C6	121.0 (7)	C14—C15—H15	120
C10—N2—Ru1	119.9 (5)	N4—C16—H16	119
C6—N2—Ru1	119.1 (5)	C17—C16—H16	119
C15—N3—C11	119.8 (7)	C16—C17—H17	120
C15—N3—Ru1	127.2 (6)	C18—C17—H17	120
C11—N3—Ru1	113.0 (5)	C17—C18—H18	121
C16—N4—C20	118.5 (6)	C19—C18—H18	121
C16—N4—Ru1	127.9 (5)	C18—C19—H19	120
C20—N4—Ru1	113.5 (5)	C20—C19—H19	120
C21—N5—C25	120.5 (7)	C21—C22—H22	121
C21—N5—Ru1	121.4 (5)	C23—C22—H22	120
C25—N5—Ru1	118.1 (5)	C23—C24—H24	121
C30—N6—C26	118.4 (7)	C25—C24—H24	121
C30—N6—Ru1	127.3 (6)	C26—C27—H27	121
C26—N6—Ru1	114.4 (5)	C28—C27—H27	121
N1—C1—C2	122.2 (7)	C27—C28—H28	120
C3—C2—C1	119.2 (7)	C29—C28—H28	121
C2—C3—C4	119.2 (7)	C28—C29—H29	120
C3—C4—C5	120.3 (7)	C30—C29—H29	120
N1—C5—C4	120.6 (7)	N6—C30—H30	119
N1—C5—C6	115.2 (6)	C29—C30—H30	119
C4—C5—C6	124.2 (7)	C31—C32—H32	180

Symmetry code: (i) −*x*+1, −*y*+2, −*z*+1.
